# Comparison of the backbone dynamics of wild-type *Hydrogenobacter thermophilus* cytochrome *c*_552_ and its *b*-type variant

**DOI:** 10.1007/s10858-015-9938-3

**Published:** 2015-05-08

**Authors:** Kaeko Tozawa, Stuart J. Ferguson, Christina Redfield, Lorna J. Smith

**Affiliations:** Department of Biochemistry, University of Oxford, South Parks Road, Oxford, OX1 3QU UK; Department of Chemistry, University of Oxford, Inorganic Chemistry Laboratory, South Parks Road, Oxford, OX1 3QR UK

**Keywords:** *C*-type cytochrome, Heme, Hydrogen exchange, ^15^N relaxation, Protein dynamics, Protein NMR

## Abstract

Cytochrome *c*_552_ from the thermophilic bacterium *Hydrogenobacter thermophilus* is a typical *c*-type cytochrome which binds heme covalently via two thioether bonds between the two heme vinyl groups and two cysteine thiol groups in a CXXCH sequence motif. This protein was converted to a *b*-type cytochrome by substitution of the two cysteine residues by alanines (Tomlinson and Ferguson in Proc Natl Acad Sci USA 97:5156–5160, 2000a). To probe the significance of the covalent attachment of the heme in the *c*-type protein, ^15^N relaxation and hydrogen exchange studies have been performed for the wild-type and *b*-type proteins. The two variants share very similar backbone dynamic properties, both proteins showing high ^15^N order parameters in the four main helices, with reduced values in an exposed loop region (residues 18–21), and at the C-terminal residue Lys80. Some subtle changes in chemical shift and hydrogen exchange protection are seen between the wild-type and *b*-type variant proteins, not only for residues at and neighbouring the mutation sites, but also for some residues in the heme binding pocket. Overall, the results suggest that the main role of the covalent linkages between the heme group and the protein chain must be to increase the stability of the protein.

## Introduction

*C*-type cytochromes are ubiquitous proteins and essential for the life of almost all organisms. They are involved in electron transport from within the cytochrome *bc*_1_ complex to cytochrome *aa*_3_ oxidase, in the nitrogen cycle of photosynthetic bacteria, as well as in the functions of enzymes such as nitrite reductase, hydroxylamine oxidoreductase and cytochrome *c* peroxidase (Barker and Ferguson 1999; Moore and Pettigrew 1990; Pettigrew and Moore 1987; Scott and Mauk 1995). It has also been reported that cytochrome *c* acts as an intermediate in apoptosis to activate caspase-9 (Liu et al. 1996).

*C*-type cytochromes are unique among the other classes of cytochromes in having covalently bound heme groups. The heme is attached to the protein at a CXXCH consensus sequence motif. The cysteine residues each form a covalent thioether bond with the two vinyl groups of the heme. This attachment does not occur spontaneously and relies on one of three types of post translational apparatus (Allen et al. 2002). There is considerable interest in trying to understand the advantage resulting from covalent attachment of heme, particularly as the majority of cytochromes do not have covalently-bound heme (Barker and Ferguson 1999; Bowman and Bren 2008; Smith et al. 2010; Stevens et al. 2004; Stevens and Ferguson 2012). In principle, several considerations could underpin the occurrence of this feature; these include enhanced stability, tuning of the redox potential, and modulation of the dynamics of the polypeptide chain.

In the case of the cytochrome *c*_552_ from *Hydrogenobacter thermophilus* (HT-*c*_552_) it has proved possible to obtain cytochrome variants with the characteristic CXXCH motif altered to AXXCH, CXXAH or AXXAH thus allowing either *c*-type cytochromes with a single thioether bond or a *b*-type cytochrome to be obtained (Tomlinson and Ferguson 2000a, b). With this system it has been found that the loss of one thioether bond has little effect on either the redox potential or the thermal stability (Tomlinson and Ferguson 2000b). In contrast, the *b*-type derivative has a redox potential lowered by 75 mV (Sambongi et al. 1989; Tomlinson and Ferguson 2000a) and is significantly less stable; wild-type HT-*c*_552_ (WT HT-*c*_552_) is highly thermostable (T_m_ = 121 °C) (Nakamura et al. 2006) but the melting temperature for the *b*-type derivative is only 58 °C (Tomlinson and Ferguson 2000a). We note here that the interpretation of stability is not straightforward in this case because the unfolding of the *b*-type cytochrome relative to the *c*-type protein is fundamentally different. In particular, there will be a greater increase in entropy when the *b*-type protein unfolds reflecting the release of the heme moiety.

Increased stability of cytochromes can be achieved by altered packing of the polypeptide chain as demonstrated by the work of Sambongi et al. who have endowed the stability of a thermophilic *c*-type cytochrome on a mesophilic one by judicious mutations (Hasegawa et al. 1999, 2000; Oikawa et al. 2005; Uchiyama et al. 2002). Sambongi et al. have also identified an even more stable *b*-type variant of a *c*-type cytochrome in their work on *Aquifex aeolicus* cytochrome *c*_555_ (Yamanaka et al. 2009, 2011). The results of studies such as these suggest that it would have been possible for a more stable version of mitochondrial cytochrome *c*, without the covalent bonds to heme, to have evolved. Yet it is notable that many mitochondrial cytochromes *c* require a biogenesis system that has evolved only for eukaryotes (Allen et al. 2002).

The redox potential of a heme group within a protein is dependent on many factors and, even with the same pair of axial ligands to the heme group, substantial variation can be achieved by changes to the immediate environment (Tai et al. 2012; Takayama et al. 2005; Worrall et al. 2007). Moreover, although *b*-type cytochromes in general do not have very high positive redox potentials, in thylakoid cytochrome *b*_559_, with bis-histidine ligation, a redox potential of +330 mV is found (Roncel et al. 2001; Stewart and Brudvig 1998). Thus, if mitochondrial cytochrome *c* needs a redox potential of +250 mV, to operate optimally, there seems no reason why a *b*-type variant with this redox potential and good stability could not have evolved.

In view of these considerations, we have investigated whether the loss of thioether bonds has an effect on the backbone dynamics in the case of HT-*c*_552_. In particular, we have used NMR spectroscopy to compare the structure and dynamics (Palmer 2004) of the wild-type and *b*-type variants of the protein. The assignment of the NMR spectrum and some characterisation of the C10A/C13A HT-*c*_552_ have been reported previously (Day et al. 2005; Pertinhez et al. 2001; Wain et al. 2001, 2004). Here, we have analysed backbone dynamics of WT HT-*c*_552_ and C10A/C13A HT-*c*_552_ and hydrogen exchange protection, along with chemical shift and coupling constant data for these variants, to try to gain insights into the role of the covalently attached heme group in *c*-type cytochromes.

## Materials and methods

### Protein expression and purification

The over-expression and purification of unlabelled recombinant HT-*c*_552_ were carried out following the methods described previously (Tomlinson and Ferguson 2000a). ^15^N-labelled protein was produced using modified M9 minimal media containing ^15^NH_4_Cl and *δ*-aminolevulinic acid as described previously (Wain et al. 2004). Protein yields were between 0.02 μmol (*b*-type mutant) and 0.04 μmol (wild-type) per litre of culture. The proteins were purified as described previously (Tomlinson and Ferguson 2000a) and the N-formyl methionine was removed using leucine aminopeptidase from *Aeromonas proteolytica* (Karan et al. 2002; Wain et al. 2004). Protein purity was confirmed by SDS-PAGE, N-terminal amino acid sequencing and mass spectrometry.

### NMR experiments and resonance assignments

NMR data were recorded on a home-built 600 MHz spectrometer located in the Department of Biochemistry, University of Oxford. The spectrometer was equipped with an Oxford Instruments Company magnet, GE/Omega software and digital control equipment, a triple-resonance pulsed field-gradient probehead, and linear amplifiers. NMR spectra were processed and analysed using the NMRPipe (Delaglio et al. 1995) and CcpNmr suites (Vranken et al. 2005).

The NMR samples contained 0.4–1 mM protein in 20 mM sodium phosphate buffer prepared with 95 % H_2_O/5 % D_2_O (with 0.1 % NaN_3_). Samples were reduced by addition of a small amount of disodium dithionite and the pH of the solution was then adjusted to 7.1. Experiments were performed at 25 °C, unless otherwise stated.

The backbone amide resonances of the wild-type protein were assigned using a 3D ^15^N-edited NOESY-HSQC experiment with a mixing time of 75 ms (Marion et al. 1989), with reference to previous assignments (Hasegawa et al. 1998; Takayama et al. 2007; Wain et al. 2004). Five of the backbone resonance assignments of the *b*-type variant were revised following further analysis of the spectra (for Gln 3, Leu 4, Lys 36, Ser 51 and Met 59). The ^1^H^N^ and ^15^N resonance assignments for the wild-type protein and the revised assignments for the *b*-type variant, plus the ^3^*J*_HNα_ coupling constants and ^15^N T_1_, T_2_ and {^1^H}–^15^N NOE values, have been deposited in the BioMagRes Bank (BMRB codes 25389 and 25390, respectively).

Backbone ^15^N T_1_ and T_2_ relaxation times and {^1^H}–^15^N heteronuclear NOE measurements were performed using spectral widths of 9090 Hz (F_2_, ^1^H) and 2000 Hz (F_1_, ^15^N). 1024 and 128 complex points were collected in F_2_ and F_1_, respectively (Kay et al. 1989). Relaxation delays for the T_1_ measurements were 40, 80, 120, 200, 400, 600, 800, 1080 and 1520 ms. Relaxation delays for the T_2_ measurements were 8.62, 17.2, 34.5, 60.4, 86.2, 138.0, 215.6 and 301.8 ms and the CPMG delay was 500 µs. The {^1^H}–^15^N heteronuclear NOE experiments were recorded in an interleaved fashion with and without ^1^H saturation for 4 s.

Amide hydrogen–deuterium exchange was monitored using a series of HSQC spectra collected over a period of ~3 days immediately after the lyophilised protein was dissolved in 99.9 % D_2_O at pH 7.3. A further HSQC was collected after 11 days. The ^3^*J*_HNα_ coupling constants were measured using an HMQC-J experiment as described previously (Kay and Bax 1990; Wain et al. 2004).

### Analysis of relaxation data

T_1_ and T_2_ were fitted as single-exponential decays to the peak intensities determined as a function of the eight or nine delay times. The heteronuclear NOE was calculated as the ratio of the peak intensities in the spectra recorded with and without ^1^H saturation. Uncertainties in the T_1_, T_2_ and {^1^H}–^15^N NOE values were estimated using baseline noise. Data analysis was carried out using the CcpNmr suite (Vranken et al. 2005).

The relaxation data for cytochrome *c*_*552*_ were analysed using an axially symmetric rotational diffusion model using the procedure described previously for human α-lactalbumin and lysozyme from bacteriophage lambda (Bruylants and Redfield 2009; Smith et al. 2013). Calculation of the T_1_/T_2_ ratio was carried out with a fixed value of S^2^ (here 0.9), an N–H bond length of 1.02 Å and a chemical shift anisotropy, (σ_∣∣_ − σ_⊥_), of −160 ppm. The 1YNR X-ray structure of *H. thermophilus* cytochrome *c*_*552*_ was used as a structural model (Travaglini-Allocatelli et al. 2005). The data for WT and C10A/C13A HT-*c*_552_ were analysed together and a single diffusion tensor determined; a D_∣∣_/D_⊥_ ratio of 1.12 was obtained from the T_1_/T_2_ data. Differences in protein concentration resulted in differences in the sample viscosity; for WT and C10A/C13A HT-*c*_552_ overall rotational correlation times, defined as (4*D_∣∣_ + 2*D_⊥_)^−1^, of 4.9 and 4.3 ns are obtained, respectively.

Relaxation data were analysed using an in-house computer program that incorporates the model-free formalism of Lipari and Szabo with Monte-Carlo error estimation (Lipari and Szabo 1982a, b; Mandel et al. 1995). The relaxation data were fitted using four models: S^2^ only (model 1), S^2^ and R_ex_ (model 2), S^2^ and τ_e_ (model 3), and S^2^, R_ex_, and τ_e_ (model 4). S^2^ is the generalised order parameter, R_ex_ is the chemical exchange contribution to T_2_ and τ_e_ is the effective correlation time for internal motion on a fast time scale. For all models, the parameters S^2^, R_ex_, and τ_e_ were optimised by minimizing the χ^2^ parameter using a downhill simplex algorithm (Johnson and Faunt 1992). The error in these parameters has been estimated from 500 Monte Carlo simulations (95 % confidence interval). Model selection was carried out as described previously using the F-test (Bruylants and Redfield 2009; Mandel et al. 1995; Smith et al. 2013). It should be noted that for model 4 the number of parameters that are fitted is equal to the number of observations and therefore the F-test for statistical significance cannot be applied. This model is only selected if the simpler models do not give a good fit to the experimental data.

## Results

### Chemical shift comparison

**Fig. 1:**
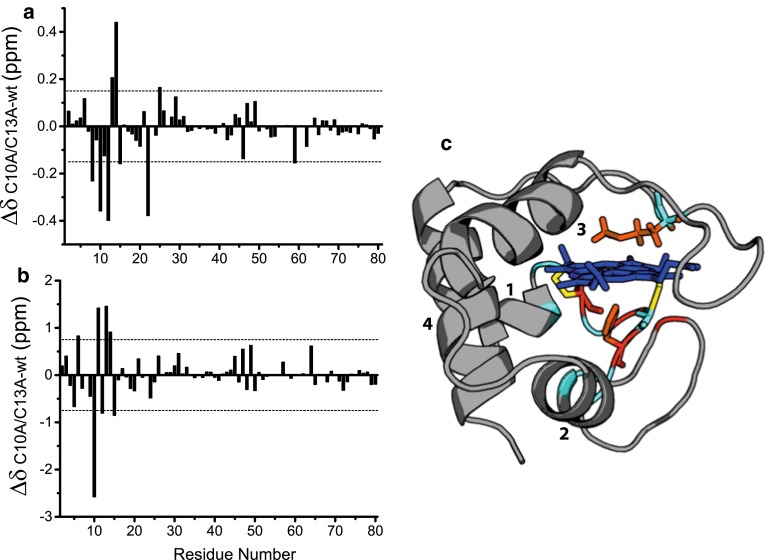
Chemical shift differences between WT and C10A/C13A HT-*c*_552_ in **a** ^1^H^N^, and **b** ^15^N observed for reduced state proteins at pH 7 and 25 °C. The ^15^N chemical shift differences have been corrected to take into account the sequence change using the ^15^N random coil chemical shift data of Braun et al. (Braun et al. 1994). *Dotted lines* show chemical shift differences of 0.15 and 0.75 ppm for ^1^H^N^ and ^15^N respectively. **c** Structure of WT HT-*c*_552_ (Hasegawa et al. 1998) with the backbone trace of residues with a combined chemical shift difference greater than 0.1 and 0.25 ppm coloured in *cyan* and *red* respectively. The heme group is shown in *blue*, the side chains of Cys10 and Cys 13 that form the thioether linkages to the heme are shown in *yellow*, the axial ligands are shown in orange and the helices (1–4) are labelled. The combined chemical shift is calculated as $\Delta \delta = \sqrt {\frac{{[\delta H^{2} + (0.102\delta N)^{2} ]}}{2}}$ (Williamson 2013). Helices 1, 2, 3 and 4 span residues 1–9, 24–33, 37–49 and 65–78, respectively

The differences between the backbone ^1^H^N^ and ^15^N amide chemical shifts of WT HT-*c*_552_ and C10A/C13A HT-*c*_552_ are shown in Fig. 1. Most of the resonances from the *b*-type mutant have similar ^1^H^N^ chemical shifts to those of the wild-type protein, as observed previously (Wain et al. 2004). ^1^H^N^ chemical shift differences greater in magnitude than 0.15 ppm are seen for Lys 8, Cys/Ala 10, Ala 12, Cys/Ala 13, His 14, Asp 15, Gly 22, Tyr 25 and Met 59. For the ^15^N chemical shifts, differences greater in magnitude than 0.75 ppm are seen for Lys 6, Cys/Ala 10, Met 11, Ala 12, Cys/Ala 13, His 14 and Asp 15. Most of the largest chemical shift differences are for the mutated residues 10 and 13 and their directly neighbouring residues in the sequence. Of the other residues that show significant chemical shift differences, the amide nitrogen atoms of Lys 6 and Lys 8 are close in space to the SG atom of Cys 10 (6.1 and 5.8 Å, respectively) and those of Asp 15 and Gly 22 are close in space to the SG atom of Cys 13 (8.7 and 5.1 Å, respectively) in the structure of WT HT-*c*_552_ (Hasegawa et al. 1998). His 14 and Met 59 are the axial ligands for the heme iron and the side chain of Tyr 25 lies adjacent to that of axial ligand His 14. The larger chemical shift differences seen for all these residues will reflect the change in environment caused by the removal of the thioether linkages and also small structural changes within the heme-binding site. The side chain of Met 59, one of the axial ligands, has significantly upfield-shifted Hβ, Hγ and Hε resonances, located between −0.5 and −4 ppm, in both WT and C10A/C13A HT-*c*_*552*_. The chemical shifts for these resonances differ by no more than 0.18 ppm between the two proteins indicating a very similar orientation of the Met 59 side chain with respect to the heme.

## ^15^N relaxation of WT HT-c_552_ and C10A/C13A HT-c_552_

**Fig. 2:**
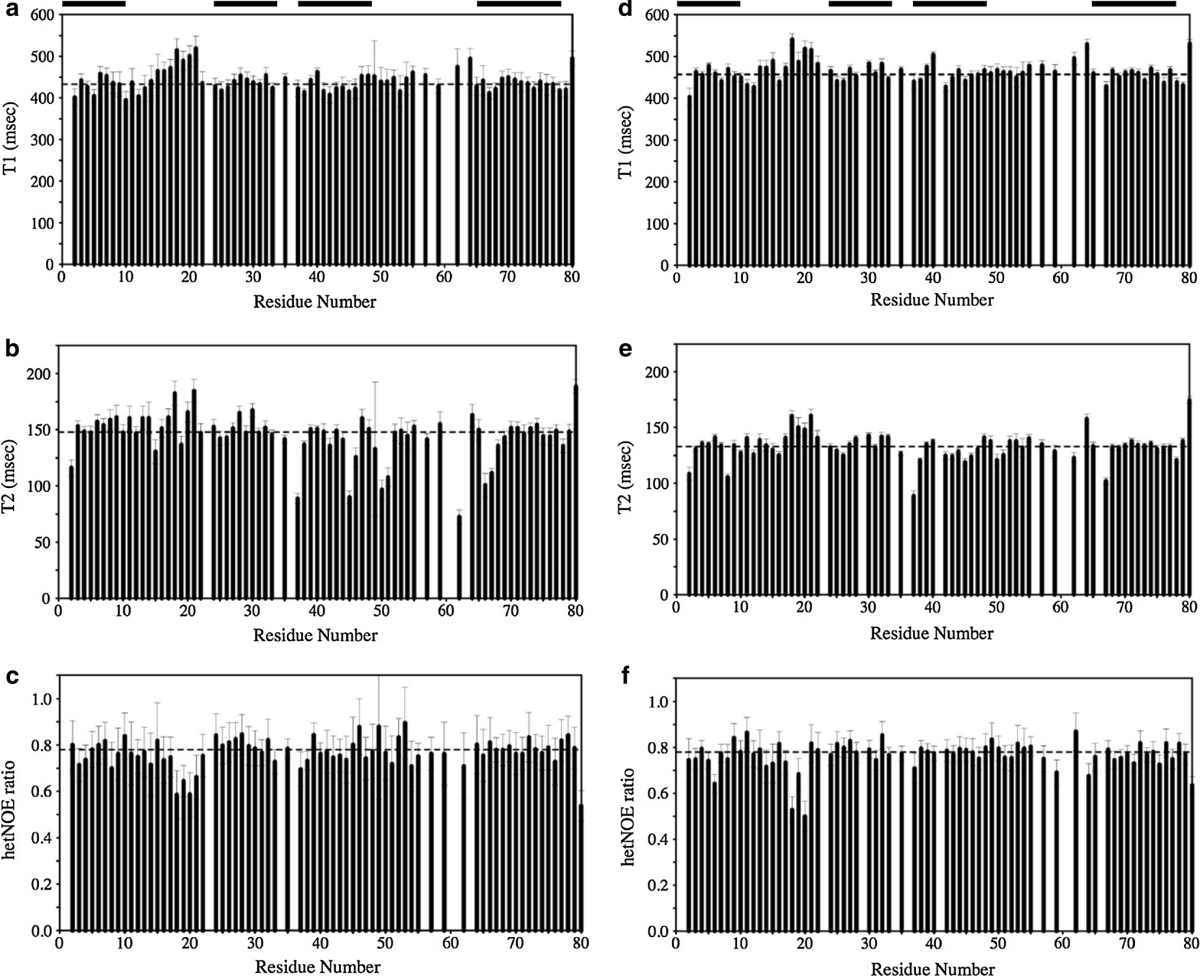
60.70 MHz ^15^N relaxation times and {^1^H}–^15^N heteronuclear NOE for backbone amides of WT HT-*c*_552_ (**a**–**c**) and C10A/C13A HT-*c*_*552*_ (**d**–**f**) in 95 % H_2_O/5 % D_2_O and 20 mM sodium phosphate buffer at pH 7.1 and 25 °C. T_1_ times (**a**, **d**), T_2_ times (**b**, **e**) and ^1^H–^15^N NOEs (**c**, **f**) are shown. The *dashed lines* show the average T_1_, T_2_ and NOE values observed for the helical core of the structures. The position of the four helices is shown schematically at the *top* of the plots

Backbone ^15^N T_1_ and T_2_ relaxation times and the {^1^H}–^15^N NOE ratios were determined for 71 residues in wild-type HT-*c*_552_ and for 68 residues in C10A/C13A HT-*c*_552_ (Fig. 2). For the wild-type protein, fairly uniform T_1_ and T_2_ values are observed across the four regions of helical structure with average T_1_ and T_2_ values of 433 ± 15 and 148 ± 10 ms, respectively. Somewhat elevated T_1_ and T_2_ values are observed for residues 18–21. In addition, the {^1^H}–^15^N NOE ratios for residues 18–21 are lower than the average of 0.78 ± 0.04 observed for the helical regions. A similar pattern of T_1_, T_2_ and {^1^H}–^15^N NOE is seen for the *b*-type variant. Residues 18–21 are located in a loop which follows the heme-attachment site and these relaxation data suggest that this loop is mobile relative to the core helical structure.

### Model-free analysis of backbone dynamics

The T_1_, T_2_ and {^1^H}–^15^N NOE values were analysed using the Lipari-Szabo model-free formalism (Lipari and Szabo 1982a, b) as described in the “Materials and methods” section. For WT HT-*c*_552_, the relaxation data for 60 of the 71 residues for which data were obtained could be fitted with a single parameter, S^2^ (model 1). For nine and one residues, two parameters, S^2^ and R_ex_ (model 2) or S^2^ and τ_e_ (model 3), respectively, gave an acceptable fit. For one residue, three parameters, S^2^, R_ex_ and τ_e_ were required (model 4). For C10A/C13A HT-*c*_552_, 57 of the 68 residues could be fitted with a single parameter, S^2^ (model 1). For seven and three residues, two parameters, S^2^ and R_ex_ (model 2) or S^2^ and τ_e_ (model 3) respectively, gave an acceptable fit. For one residue, three parameters, S^2^, R_ex_ and τ_e_ were selected (model 4).

**Fig. 3:**
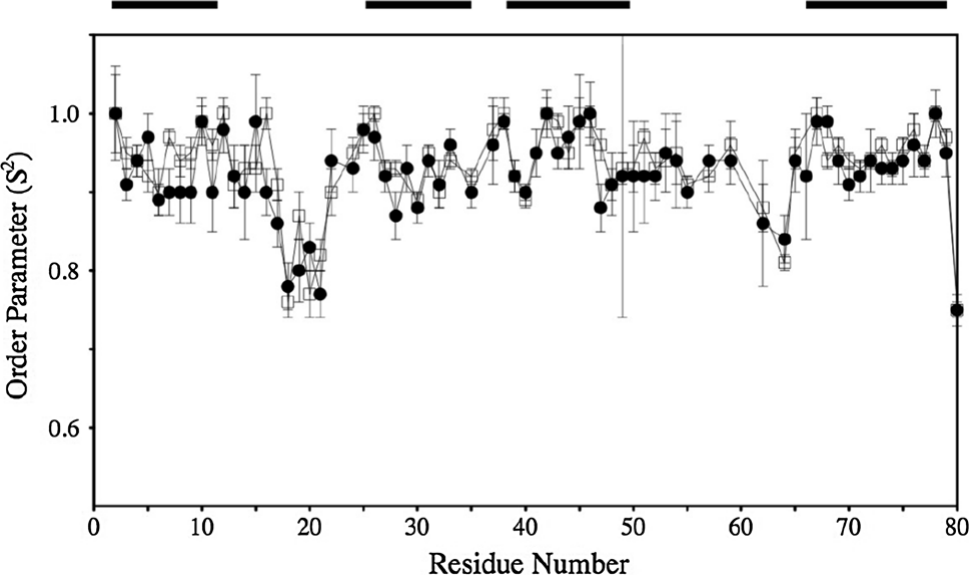
Order parameters, S^2^, obtained from the fitting of the ^15^N relaxation data for WT HT-*c*_552_ (*filled circles*) and C10A/C13A HT-*c*_552_ (*open squares*) at pH 7.1 and 25 °C. The position of the four helices is shown schematically at the top of the plot

The majority of residues in both variants show order parameters, S^2^, between 0.85 and 1.0, indicating restricted mobility of the backbone (Fig. 3). In the helical regions, average S^2^ values of 0.94 ± 0.04 and 0.95 ± 0.03 are observed for WT HT-*c*_552_ and C10A/C13A HT-*c*_552_, respectively. The loop that follows helix 1 contains the CXXCH/AXXAH motif including one of the axial ligands (His 14). In both proteins these residues (10–17) have average order parameters that are the same as the helical core. Thus, removal of the covalent bonds between the heme and residues 10 and 13 does not lead to an enhancement in fast timescale dynamics in this region of the structure. The S^2^ values of residues 18–21, which precede helix 2, are lower for both variants indicating higher mobility than observed for the helical core. The increased dynamics in this loop is in agreement with MD simulation results. Residues in this region show elevated root-mean-square backbone atom fluctuations (RMSF) in simulations of WT HT-*c*_552_ and C10A/C13A HT-*c*_552_ performed at 298 K (Smith et al. 2006). Lower order parameters are also observed in both variants for residues 62 and 64, immediately preceding helix 4, and the C-terminal residue, Lys 80. The loop connecting helices 3 and 4 contains the second axial ligand, Met 59. S^2^ values similar to those of the helical core are observed for residues 50–59 in both WT HT-*c*_552_ and C10A/C13A HT-*c*_552_. In summary, the conversion of this cytochrome from *c*-type to *b*-type does not result in any change in the fast timescale dynamics of the protein backbone. The same conclusion was drawn previously from comparisons of MD simulations of WT HT-*c*_552_ and C10A/C13A HT-*c*_552_, the two proteins showing very similar backbone torsion angle fluctuations at 298 K (Smith et al. 2006).

## ^3^*J*_HNα_ coupling constants

**Fig. 4:**
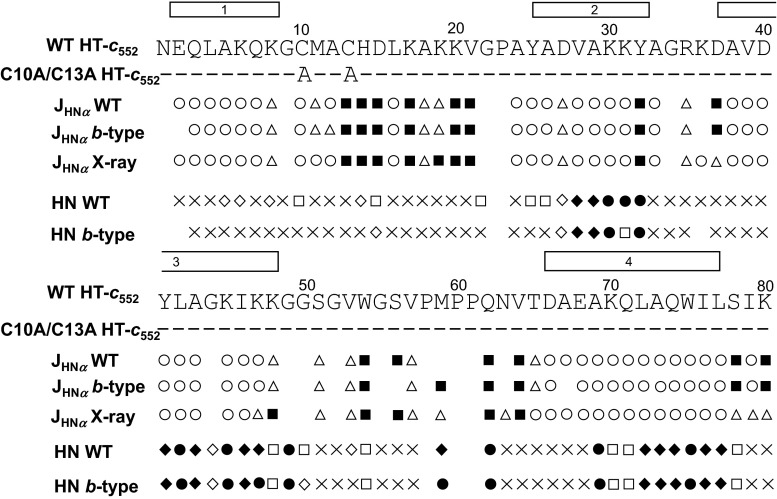
Summary of the amide hydrogen–deuterium exchange data for reduced WT HT-*c*_552_ and C10A/C13A HT-*c*_552_ at pH 7, 25 °C are indicated along with the amino acid sequences of the proteins. A *cross* indicates backbone sites that exchange completely within the first hour; *open diamond* and *open square* indicate sites that exchange between 1 and 10 h and between 10 and 67 h, respectively; *filled circle* indicates backbone sites that are not fully exchanged after 67 h; *filled diamond* indicates sites that are not exchanged after 11 days. Summaries of the ^3^ *J*_HNα_ coupling constant values for WT HT-*c*_552_ and C10A/C13A HT-*c*_552_ are also shown together with ^3^ *J*_HNα_ values predicted from the ϕ angles in the X-ray structure of WT HT-*c*_552_ (Travaglini-Allocatelli et al. 2005). Coupling constants larger than 8 Hz, between 8 and 6 Hz inclusive and less than 6 Hz are indicated by *filled square*, *open triangle* and *open circle*, respectively. The positions of the four α-helices in the X-ray structure of WT HT-*c*_552_ are shown by *open bars* above the protein sequence

^3^*J*_HNα_ coupling constants of each residue of WT HT-*c*_552_ were measured and compared to the data of the *b*-type protein reported previously (Wain et al. 2004) (Fig. 4). The overall values of the coupling constants for the two variants are very similar; of the 61 residues for which the ^3^*J*_HNα_ values could be measured in both proteins, 60 of the residues have coupling constant values in the same category (less than 6 Hz, in the range 6–8 Hz or greater than 8 Hz) in WT HT-*c*_552_ and C10A/C13A HT-*c*_552_. This close similarity suggests that the mutations do not lead to significant changes in the backbone conformation.

Ala 12 shows a larger difference in the ^3^*J*_HNα_ value between the two proteins. Here the ^3^*J*_HNα_ value is 5.2 Hz in WT HT-*c*_552_ and 6.5 Hz in C10A/C13A HT-*c*_552_. A comparison of the experimental ^3^*J*_HNα_ values with those predicted from the ϕ torsion angles in the 1YNR X-ray structure of WT HT-*c*_552_ (Travaglini-Allocatelli et al. 2005) shows that the experimental coupling constants for Ala 12 in both proteins are larger than that predicted from the X-ray structure (ϕ −58.5°; predicted ^3^*J*_HNα_ 4.0 Hz). This is also the case for Met 11 where the X-ray structure predicted ^3^*J*_HNα_ value is 4.9 Hz but the experimental ^3^*J*_HNα_ values for both WT HT-*c*_552_ and C10A/C13A HT-*c*_552_ are 7.5 and 7.0 Hz, respectively. Met 11 and Ala 12 lie between the two residues (Cys 10 and Cys 13) that make thioether linkages to the heme group in WT HT-*c*_552_. The differences in experimental coupling constant values of the two variants and between the experimental coupling constants and those predicted from the X-ray structure suggest that there might be some conformational flexibility for this region in solution and that the level of the dynamics may increase on the loss of the covalent linkages to the heme group in the *b*-type variant.

Comparisons of the ^3^*J*_HNα_ values predicted from the 1YNR X-ray structure of WT HT-*c*_552_ with the experimental coupling constants for the rest of the sequence shows good agreement. For 53 of the 60 residues that have coupling constant values in the same category in WT HT-*c*_552_ and C10A/C13A HT-*c*_552_, the predicted coupling constant is also in the same category. Residues 18–21 are located in a loop preceding helix 2. ^15^N relaxation has shown enhanced fast timescale dynamics for these residues. Good agreement is found between the experimental and predicted coupling constants for residues 18, 20 and 21. For Lys 19 both the experimental coupling constants are ~6 Hz while the value calculated from the X-ray structure is 8.3 Hz. This loop is involved in crystal contacts in the X-ray structure. There are also larger differences at the C-terminus suggesting some change to the backbone conformation in solution compared to the crystal structure. In particular, in both WT HT-*c*_552_ and C10A/C13A HT-*c*_552_, Ser 78 and Lys 80 have experimental ^3^*J*_HNα_ values greater than 8 Hz (8.2/8.0 Hz and 8.4/8.4 Hz) and Ile 79 has experimental ^3^*J*_HNα_ values less than 6 Hz (5.3/5.4 Hz). In contrast, the ^3^*J*_HNα_ values predicted for all three residues from the X-ray structure ϕ angles are 6.7–6.8 Hz. Interestingly, there is also a difference for Met 59, one the axial ligands to the heme, between the experimental ^3^*J*_HNα_ value in the *b*-type variant (>8 Hz) and the ^3^*J*_HNα_ value predicted from the X-ray structure ϕ angle (7.1 Hz). The experimental ^3^*J*_HNα_ value could not be measured for WT HT-*c*_552_ due to partial resonance overlap.

### Hydrogen–deuterium exchange of backbone amide protons

Hydrogen–deuterium exchange for backbone amide protons in WT HT-*c*_552_ and C10A/C13A HT-*c*_552_ was monitored by a series of HSQC experiments over 67 h and then an experiment 11 days after the proteins were dissolved in deuterium oxide (Fig. 4). These hydrogen exchange data have been compared with the hydrogen bonds present in the 1YNR X-ray structure of WT HT-*c*_552_ (Travaglini-Allocatelli et al. 2005). WT and C10A/C13A HT-*c*_552_ have very similar patterns of hydrogen exchange, with 21 and 20 slowly exchanging backbone amide protons respectively (resonances present after 67 h or longer). All but two of the residues with slowly exchanging amide protons are involved in NH(i)–CO(i-4) hydrogen bonds in helices 2, 3 and 4 in the crystal structure of the wild-type protein. The protection patterns for these residues are closely similar in the two proteins. In helix 1 much lower levels of hydrogen exchange protection are seen, and there are also differences between the protection patterns in the two proteins. In particular, the amide protons of Ala 5, Lys 6 and Lys 8 are more protected in the wild-type protein than the *b*-type variant. The faster hydrogen exchange in the *b*-type variant may reflect greater conformational dynamics in the absence of the covalent linkages between the heme group and the protein. Another contribution may be an increase in the small population of unfolded conformers present in solution for C10A/C13A HT-*c*_552_, in agreement with the observed lower thermal stability of the *b*-type variant compared to WT HT-*c*_552_.

Similar data were seen in MD simulations of WT and C10A/C13A HT-*c*_552_ (Smith et al. 2006), with highly persistent hydrogen bonds in helices 2, 3 and 4, and much lower hydrogen bond populations with differences between the two proteins in helix 1. The simulations also show some fluctuating population of NH(i)–CO (i-4) helical-type hydrogen bonds for residues in the region following helix 1 (residues 9–15). Experimentally the amide protons in this region show some hydrogen exchange protection, with slightly reduced protection in the double alanine mutant. The observed hydrogen exchange protection may therefore come from the population of fluctuating helical hydrogen bonds, with differences reflecting the mutations in the CXXCH motif in the *b*-type variant.

There are two amide protons that are not in helical regions of the protein but are slowly exchanging in the wild-type protein and *b*-type variant (Met 59 and Gln 62). Both of these residues are in the loop region between helices 3 and 4 in WT HT-*c*_552_ and their amide protons are involved in hydrogen bonds in the crystal structure (59NH–50CO and 62NH–47CO). Gly 50 is also involved in a hydrogen bond linking the same regions of the sequence (50NH–59CO) and its amide proton has a moderate level of protection (though this is reduced in the *b*-type variant). This region of the protein is of particular interest as residues from it form part of the heme-binding site (Met 59 is an axial ligand). Two other residues in this region have amide protons that show moderate levels of hydrogen exchange protection (Val 53 and Trp 54). The amide protons of the residues form hydrogen bonds to the heme propionate groups in the crystal structure. The level of protection is reduced for Val-53 in the *b*-type variant compared to the wild-type protein suggesting that there are some changes to the persistence of these hydrogen bonds in the *b*-type variant.

Another area where there are some subtle differences in hydrogen exchange protection is at the start of helix 2. Moderate levels of hydrogen exchange protection are seen for residues 25–27 at the start of this helix in the wild-type protein with the level of protection being reduced in the *b*-type variant. The amide protons of all these three residues form hydrogen bonds to the backbone carbonyl groups of residues in the region connecting helices 1 and 2 in the X-ray structure. Tyr 25 and Ala 26 also have low solvent accessibilities. Reductions in hydrogen exchange protection in the *b*-type variant are also seen for His 14, Asp 15 and Gly 22 in this region suggesting that the Cys to Ala mutations, and loss of covalent links to the heme group, within the part of the sequence may have increased the dynamics or the solvent accessibility of the protein backbone. We note that His 14, Asp 15, Gly 22 and Tyr 25 all also show significant changes in ^1^H^N^ and/or ^15^N chemical shift values between the wild-type protein and the *b*-type variant.

## Discussion

The ^15^N relaxation and hydrogen exchange data for HT-*c*_552_ reported in this paper show that the α-helices in the protein form a rigid framework whose backbone amide groups exhibit high order parameters and high levels of hydrogen exchange protection (for helices 2, 3 and 4). The lowest order parameters are seen in the loop region between helices 1 and 2 and at the C-terminus. Similar results have been reported for a number of other proteins adopting the α-helical fold of class I *c*-type cytochromes, especially when the iron is in its reduced state (Banci et al. 2002; Barker et al. 2001; Bartalesi et al. 2003; Cordier et al. 1998; Fetrow and Baxter 1999; Liu et al. 2009; Russell et al. 2003; Ubbink et al. 1996). In general, any significant fast timescale backbone motions are restricted to exposed residues in loop regions and the protein termini, with the four helices forming a rigid core. For example, for *T. versutus* cytochrome *c*_550_ in the reduced state, high order parameters are seen in the four helices with lower order parameters in the loop regions between helices 1 and 2 and between helices 4 and 5; this protein also has an elongated C-terminal tail which is highly mobile (Ubbink et al. 1996). Similarly, for reduced mitochondrial cytochrome *c*, the greatest mobility on the picosecond to nanosecond timescale is seen in the loop B/C region (Fetrow and Baxter 1999).

Our results show that there are only very subtle changes to the structure and dynamics of HT- *c*_552_ on removal of the thioether linkages forming the *b*-type variant. There are no significant differences between the ^15^N relaxation data for wild-type cytochrome *c*_552_ and the *b*-type variant suggesting no change in fast timescale backbone dynamics upon removal of the covalent bonds to the heme. However, in addition to chemical shift changes observed for the mutated residues and those directly adjacent to them in the sequence, some small differences in hydrogen exchange protection and/or chemical shifts are seen. These include changes for Lys 6 and Lys 8 in helix 1, Gly 22 and Tyr 25 in the loop between helices 1 and 2, His 14 and Met 59 which form the axial ligands to the heme group and Val 53, which forms hydrogen bonds to a heme propionate group in the X-ray structure WT HT-*c*_552_. All these residues are in the vicinity of the heme-binding pocket and the differences may reflect changes in out-of-plane deformations of the heme group (Kleingardner et al. 2013; Sun et al. 2014). The changes for the axial ligand His 14 could also result from changes in the His-Fe interaction, a factor which has been suggested to play a role in tuning heme redox potentials (Bowman and Bren 2008; Michel et al. 2007), or changes in the CXXCH loop stiffness (Galinato et al. 2015). However, the magnitude of the differences observed suggests that any changes in structure and dynamics of the protein are small.

Other studies where there have been changes to the covalent bonds between the heme group and the protein also report only minor differences in the protein structure or dynamics. As for HT-*c*_552_ any differences are normally concentrated in the vicinity of the heme group and cross-links. For example, the ^15^N relaxation and hydrogen exchange protection of *Synechococcus* sp. PCC 7002 hemoglobin has been studied in the presence and absence of a cross-link between the heme 2-vinyl group and His 117 (Pond et al. 2012; Vuletich et al. 2006). The ^15^N order parameters and hydrogen exchange data of both proteins are closely similar. However, some differences are seen in T_2_ values and increased protection is observed in the protein with the cross linkage near to His 117 itself and to His 70, the proximal heme ligand. For mitochondrial cytochrome *c*, the C14S variant adopts the wild-type structure but shows increased mobility in the region of Trp 59, adjacent to the heme binding site, compared to the wild-type protein (Rosell and Mauk 2002). In the case of cytochrome *b*_562_ variants have been studied which have one or two covalent bonds between the heme and cysteine residues engineered into the protein sequence (Arnesano et al. 2000; Assfalg et al. 2001; Faraone-Mennella et al. 2006). These variants have very similar structures to the wild-type protein but ^15^N relaxation studies of R98C cytochrome *b*_562_, which has one thioether linkage between the heme and protein, show increased dynamics for residues close to the heme-binding site compared to the wild-type protein (Assfalg et al. 2001). The authors suggest that this increase in mobility may be due to strain in the structure resulting from the artificial covalent bond.

Although the changes to the structure or dynamics of these proteins on introduction or loss of the protein-heme covalent linkages are limited, there are striking differences in the protein stability. In all cases the proteins with covalent linkages show higher stability to thermal or chemical denaturation. For C10A/C13A HT-*c*_552_ studied here, the *b*-type variant has a melting temperature (T_m_) of 58 °C compared to the wild-type protein which has a T_m_ of 121 °C (Nakamura et al. 2006; Tomlinson and Ferguson 2000a), although the single thioether bond variants, AXXCH and CXXAH, have similar stabilities to the wild-type protein (Tomlinson and Ferguson 2000b). *Hydrogenobactor thermophilus* lives in hot springs at temperatures of 70–75 °C. So, although the *b*-type variant is stable under laboratory conditions, it would be largely unfolded under the conditions in which the bacterium grows (Kawasumi et al. 1984). Similarly, for *Aquifex aeolicus* cytochrome *c*_555_, the *b*-type variant has a melting temperature of 79 °C compared to 130 °C for the wild-type *c*-type protein (Yamanaka et al. 2009). Again, *Aquifex aeolicus* lives in hot springs at temperatures of 85–95 °C and so greater than the melting temperature of the *b*-type variant (Deckert et al. 1998). For *Synechococcus* sp. PCC 7002 hemoglobin the melting temperatures from thermal denaturation studies are 76.4 and >95 °C for the protein without and with the covalent cross-link (Vuletich et al. 2006) and for cytochrome *b*_562_, the folding free energy changes, determined from GuHCl denaturation experiments, are −30, −35.6 and −42 kJ mol^−1^ for the wild-type protein, the R98C mutant and the R98C/Y101C double mutant respectively (Faraone-Mennella et al. 2006). Yeast iso-1-cytochrome *c* has a T_m_ of 60 °C (Liggins et al. 1999). If conversion of this protein to a *b*-type cytochrome leads to a decrease in T_m_ of 20–30 °C then this protein would only be marginally stable under ‘native’ conditions. All these results suggest that one of the main driving forces for nature including covalent linkages between the heme group and the protein chain must be to increase the stability of the proteins involved, and that the alternative of increasing stability by altering the amino acid sequence, as has been done in vitro for *P. aeruginosa* cytochrome *c*_551_ (Uchiyama et al. 2002), has not occurred in vivo for molecules such as mitochondrial cytochrome *c*.

## Abbreviations

C10A/C13A HT-*c*_552_: *B*-type variant of cytochrome *c*_552_ from *Hydrogenobacter thermophilus*
GuHCl: Guanidine hydrochloride
HMQC: ^1^H–^15^N heteronuclear multiple-quantum correlation
HSQC: Heteronuclear single-quantum correlation
HT-*c*_552_: Cytochrome *c*_552_ from *Hydrogenobacter thermophilus*
MD: Molecular dynamics
NMR: Nuclear magnetic resonance
NOE: Nuclear Overhauser effect
ppm: Parts per million
WT HT-*c*_552_: Wild-type cytochrome *c*_552_ from *Hydrogenobacter thermophilus*
